# Pro‐arrhythmic atrial phenotypes in incrementally paced murine *Pgc1β* ^−/−^ hearts: effects of age

**DOI:** 10.1113/EP086589

**Published:** 2017-10-29

**Authors:** Haseeb Valli, Shiraz Ahmad, James A. Fraser, Kamalan Jeevaratnam, Christopher L.‐H. Huang

**Affiliations:** ^1^ Physiological Laboratory University of Cambridge Cambridge UK; ^2^ PU‐RCSI School of Medicine Perdana University Serdang Selangor Darul Ehsan Malaysia; ^3^ Faculty of Health and Medical Sciences University of Surrey Guildford UK; ^4^ Department of Biochemistry University of Cambridge Cambridge UK

**Keywords:** peroxisome proliferator activated receptor‐γ coactivator‐1 (PGC‐1), atria, action potential, cardiac arrhythmia

## Abstract

**New Findings:**

- **What is the central question of this study?**
  Can we experimentally replicate atrial pro‐arrhythmic phenotypes associated with important chronic clinical conditions, including physical inactivity, obesity, diabetes mellitus and metabolic syndrome, compromising mitochondrial function, and clarify their electrophysiological basis?
- **What is the main finding and its importance?**
  Electrocardiographic and intracellular cardiomyocyte recording at progressively incremented pacing rates demonstrated age‐dependent atrial arrhythmic phenotypes in Langendorff‐perfused murine *Pgc1β* ^−/−^ hearts for the first time. We attributed these to compromised action potential conduction and excitation wavefronts, whilst excluding alterations in recovery properties or temporal electrophysiological instabilities, clarifying these pro‐arrhythmic changes in chronic metabolic disease.

Atrial arrhythmias, most commonly manifesting as atrial fibrillation, represent a major clinical problem. The incidence of atrial fibrillation increases with both age and conditions associated with energetic dysfunction. Atrial arrhythmic phenotypes were compared in young (12–16 week) and aged (>52 week) wild‐type (WT) and peroxisome proliferative activated receptor, gamma, coactivator 1 beta (Ppargc1b)‐deficient (*Pgc1β* ^−/−^) Langendorff‐perfused hearts, previously used to model mitochondrial energetic disorder. Electrophysiological explorations were performed using simultaneous whole‐heart ECG and intracellular atrial action potential (AP) recordings. Two stimulation protocols were used: an S1S2 protocol, which imposed extrasystolic stimuli at successively decremented intervals following regular pulse trains; and a regular pacing protocol at successively incremented frequencies. Aged *Pgc1β* ^−/−^ hearts showed greater atrial arrhythmogenicity, presenting as atrial tachycardia and ectopic activity. Maximal rates of AP depolarization (d*V*/d*t*_max_) were reduced in *Pgc1β* ^−/−^ hearts. Action potential latencies were increased by the *Pgc1β* ^−/−^ genotype, with an added interactive effect of age. In contrast, AP durations to 90% recovery (APD_90_) were shorter in *Pgc1β* ^−/−^ hearts despite similar atrial effective recovery periods amongst the different groups. These findings accompanied paradoxical decreases in the incidence and duration of alternans in the aged and *Pgc1β* ^−/−^ hearts. Limiting slopes of restitution curves of APD_90_ against diastolic interval were correspondingly reduced interactively by *Pgc1β* ^−/−^ genotype and age. In contrast, reduced AP wavelengths were associated with *Pgc1β* ^−/−^ genotype, both independently and interacting with age, through the basic cycle lengths explored, with the aged *Pgc1β* ^−/−^ hearts showing the shortest wavelengths. These findings thus implicate AP wavelength in possible mechanisms for the atrial arrhythmic changes reported here.

## Introduction

Arrhythmogenesis is a complex physiological phenomenon of dysregulated cardiac electrical activity, with both short‐ and long‐term consequences. The complex embryological origins and anatomical structure of the atria make them particularly vulnerable to arrhythmic syndromes. Atrial fibrillation (AF) is of particular clinical importance, affecting 1–3% of individuals in Western countries (Friberg & Bergfeldt, 2013). The processes underpinning its induction and maintenance remain incompletely explained, but involve complex interactions between altered cardiac electrical properties and functional changes in the atria, which occur over the short and long term. It has been suggested that AF is a self‐perpetuating process, triggered initially by focal ectopic activity arising in the pulmonary veins that drive cumulative electrical and structural remodelling processes, themselves generating arrhythmic substrate (Haïssaguerre *et al*. 1998).

These changes are exacerbated by several interacting upstream factors, with ageing and metabolic disease central to a number of these. There is a pronounced increase in the prevalence of AF with age, from ∼4% of individuals aged 60–70 years to nearly 20% of individuals ≥80 years (Zoni‐Berisso *et al*. 2014). Likewise, metabolic factors may explain ∼60% of current upward trends in incidences of AF (Miyasaka *et al*. 2006). Metabolic disease and obesity have been implicated as risk factors, themselves age dependent, for ventricular arrhythmias (Adabag *et al*. 2015). Likewise, the risk of AF increases with physical inactivity (Mozaffarian *et al*. 2008), obesity (Tedrow *et al*. 2010), diabetes mellitus (Nichols *et al*. 2009) and metabolic syndrome (Watanabe *et al*. 2008). Amelioration of metabolic disease improves both risk profiles and responses to therapy (Tedrow *et al*. 2010). Furthermore, it has been shown that manipulation of key components of cellular energy production pathways suppresses arrhythmia in known arrhythmogenic models (Liu *et al*. 2009).

Mitochondrial function may be integral to relationships between ageing, metabolism and arrhythmia. Mitochondria provide >95% of the ATP required for Ca^2+^ homeostasis and maintenance of transmembrane ionic gradients in addition to cardiac muscle contraction (Barth & Tomaselli, 2009). Abnormal mitochondrial structure and function have been reported in animal models of AF (Ausma *et al*. 1997). Analysis of mitochondria from cardiomyocytes of human AF patients demonstrates increased DNA damage (Tsuboi *et al*. 2001; Lin *et al*. 2003), structural abnormalities (Bukowska *et al*. 2008) and impaired function (Lin *et al*. 2003). Furthermore, a number of targeted mitochondrial DNA mutations accumulate with age and show increased incidences in AF (Lai *et al*. 2003). Mitochondrial dysfunction also results in generation of reactive oxygen species, which has been implicated in the pathogenesis of human AF (Korantzopoulos *et al*. 2007). Experiments producing acute mitochondrial impairment through ischaemia–reperfusion correspondingly demonstrate pro‐arrhythmic regional heterogeneities in ventricular action potential duration (APD) and arrhythmogenesis suppressed by pharmacological manipulations of the mitochondrial membrane potential (Akar *et al*. 2005; see also Brown *et al*. 2010). These pro‐arrhythmic changes were successfully suppressed by pharmacological manipulations of the mitochondrial membrane potential (Akar *et al*. 2005). Further work demonstrated that stabilization of the mitochondrial membrane potential also inhibits arrhythmias in other settings (Brown *et al*. 2010).

However, few experiments have explored the effects of chronic age‐dependent energetic deficiency arising from ageing and mitochondrial dysfunction on the generation of atrial arrhythmias or determined their underlying electrophysiological abnormalities. The present study used a peroxisome proliferative activated receptor, gamma, coactivator 1 beta (Ppargc1b; hereafter Pgc1β)‐deficient (*Pgc1β* ^−/−^) model (Mouse Genome Informatics identification: MGI:2444934 (http://www.informatics.jax.org/)) that has been used previously to study the biochemical consequences of metabolic energetic deficiency. This family of transcriptional coactivators provides central regulators of cellular and mitochondrial function controlling key metabolic pathways, including fatty acid oxidation, mitochondrial biogenesis and oxidative phosphorylation. It occurs abundantly in energetically active tissue, such as the heart (Lin *et al*. 2005). Reduced Pgc1 levels and impaired mitochondrial function occur in diabetes mellitus, the metabolic syndrome, ageing and heart failure (Garnier *et al*. 2003; Leone & Kelly, 2011). Despite normal baseline function, *Pgc1β‐*deficient mice demonstrate compromised heart rate responses with adrenergic stimulation (Lelliott *et al*. 2006). Their *ex vivo* Langendorff‐perfused hearts show ventricular arrhythmic tendencies, abnormal diastolic Ca^2+^ transients and altered ion channel expression (Gurung *et al*. 2011). However, neither their atrial arrhythmic phenotypes nor their accompanying electrophysiological changes have been explored.

The present study complements previous reports in murine hearts carrying genetic abnormalities in specific ion channels modelling ventricular arrhythmic conditions (Huang, 2017). The arrhythmic substrate in these different exemplars was variously identified with altered action potential (AP) initiation and conduction (Martin *et al*. 2011; Ning *et al*. 2016), AP recovery (Sabir *et al*. 2007b) and arrhythmic triggers (Thomas *et al*. 2007; Goddard *et al*. 2008). The present experiments, likewise, looked for the presence of arrhythmic phenotypes provoked by the imposition of extrasystolic S2 stimuli at differing S1S2 intervals following trains of regular S1 pacing as well as steady‐state pacing at progressively decreased basic cycle lengths (BCLs). The findings were then matched to results from simultaneous determinations of AP activation and recovery, as well as temporal instabilities in the form of AP alternans (Matthews *et al*. 2010), APD–diastolic interval (DI) restitution relationships (Kim *et al*. 2002; Matthews *et al*. 2012) and spatiotemporal indicators of AP wavelength. This study represents the first to make such measurements in murine atria. Studies in human AF have reported increased slopes in restitution plots of APD–DI, intervening between full AP recovery and the peak of the subsequent AP. However, these studies did not look for accompanying alternans (Kim *et al*. 2002). However, the present findings do support previous reports associating AF with paradoxical suppression of APD alternans in a vagally mediated canine AF model (Lu *et al*. 2011).

## Methods

### Animals and ethical approval

This research was regulated under the Animals (Scientific Procedures) Act 1986 Amendment Regulations 2012 following ethical review by the University of Cambridge Animal Welfare and Ethical Review Body (Home Office PPL no. 70/8726), and followed recommendations provided by the Physiological Society (Grundy, 2015). Mice were housed in an animal facility with 12 h–12 h light–dark cycles at a temperature maintained at 21°C. Animals were fed sterile chow (RM3 Maintenance Diet; SDS, Witham, UK) and had free access to water, bedding and environmental stimuli. The C57/B6 mice strain was used as the background for both wild‐type (WT) and *Pgc1β* ^−/−^ mice, generated using a triple LoxP targeting vector as previously described (Lelliott *et al*. 2006). Four experimental groups were studied: young WT (*n* = 20), young *Pgc1β* ^−/−^ (*n* = 23), aged WT (*n* = 22) and aged *Pgc1β* ^−/−^ (*n* = 22). All young mice were aged between 12 and 16 weeks and aged mice >52 weeks. Mice were administered 200 IU of unfractionated heparin (Sigma‐Aldrich, Poole, UK) in the intraperitoneal space before being killed by cervical dislocation [Schedule 1; Animals (Scientific Procedures) Act 1986]. No recovery, anaesthetic or surgical procedures were required.

### Buffering media

Experimental solutions were based on a Krebs–Henseleit (KH) solution of the following composition (mm): NaCl, 119; NaHCO_3_, 25; KCl, 4.0; KH_2_PO_4_, 1.2; MgCl_2_, 1.0; CaCl_2_, 1.8; glucose, 10; and sodium pyruvate, 2.0; pH adjusted to 7.4 and bubbled with 95% O_2_–5% CO_2_ (British Oxygen Company, Manchester, UK). Chemical agents were purchased from Sigma‐Aldrich (Poole, UK) unless otherwise indicated. Before perfusion of isolated Langendorff‐perfused hearts with plain KH solution, perfusion was initiated in the presence of 20 μm blebbistatin (Selleckchem, Newmarket, UK) to reduce movement and allow stable impalements.

### Whole‐heart intracellular microelectrode recordings

Animals were killed for rapid sternectomy and cardiectomy. Hearts were cannulated and secured as previously described (Zhang *et al*. 2011; Matthews *et al*. 2012; Ning *et al*. 2016). No obvious macroscopic defects were observed in any heart. Hearts were then mounted on a horizontal Langendorff apparatus that was electrically insulated and incorporated into an intracellular rig within a Faraday cage, incorporating a light microscope (objective ×5, eyepiece ×5; W. Watson and Sons Limited, London, UK), organ bath, custom‐built microelectrode amplifier and head stage.

To facilitate impalement of the left atrium, hearts were mounted in a standard anatomical position to allow pacing from the right atrium (RA) and access to the left atrium (LA). The left atrium was displaced posteriorly and held in place with three A1 insect pins. The positions of the recording and stimulating electrodes were controlled by two precision micromanipulators (Prior Scientific Instruments, Cambridge, UK). In all experiments, the stimulating electrode was consistently positioned at the posterior aspect of the RA, and recordings were made from the central region of the LA, minimizing variability in distances between the respective electrodes, so that alterations in AP latencies therefore provided indications of corresponding conduction velocity changes. Hearts were perfused with plain KH solution (flow rate of 2.05 ml min^−1^) to allow establishment of a regular intrinsic rhythm. Preparations with an intrinsic rate of <5 Hz or which did not display 1:1 atrioventricular conduction for >10 min postperfusion were not used for experimentation. Perfusion with KH containing 20 μm blebbistatin was then commenced until motion was adequately minimized before resumption of perfusion with plain KH solution.

A microelectrode (tip resistance 15–25 MΩ) was pulled by a custom‐built microelectrode puller from a glass pipette (1.2 mm o.d., 0.69 mm i.d.; Harvard Apparatus, Cambourne, UK), filled with 3 m KCl and then inserted into a right‐angled microelectrode holder (Harvard Apparatus). The recorded signal from the microelectrode was passed through a head stage for preamplification forming part of a high‐input impedance direct‐current microelectrode amplifier system (University of Cambridge, Cambridge, UK) before bandpass filtering (between 0 and 2 kHz) and analog‐to‐digital conversion at a sampling frequency of 10 kHz (1401; Cambridge Electronic Design, Cambridge, UK). A successful impalement was identified by the abrupt appearance of a resting membrane potential more negative than −70 mV and regular APs with a stable waveform. A Ag/AgCl electrode in contact with the bath solution was used as the reference electrode for all measurements.

### Whole‐heart ECG recordings

Correlation between electrical signals at the level of the whole organ and intracellular voltage recordings was permitted by simultaneous ECG recordings from the Langendorff‐perfused hearts. Two unipolar ECG electrodes were placed at fixed positions adjacent to the heart in the organ bath that corresponded to the standard three‐lead ECG co‐ordinates. The recorded signals were passed through head stages for preamplification before amplification (Neurolog NL104 amplifier), bandpass filtering (between 5 and 500 Hz; NL 125/6 filter; Digitimer, Welwyn Garden City, UK) and digitization at a sampling frequency of 10 kHz (1401; Cambridge Electronic Design).

### Pacing protocols

Hearts were stimulated at an amplitude of twice diastolic threshold voltage plus 0.5 mV. Hearts underwent two separate pacing protocols during each experiment. First, a standardized S1S2 protocol was used to determine the atrial effective refractory period (AERP) from the ECG recordings. This delivered successive trains of eight S1 stimuli separated by an interval of 125 ms, followed by a solitary extrasystolic stimulus (S2) initially delivered 89 ms after the preceding S1 stimulus. This pattern of stimulation was repeated with the S1S2 interval decremented by 1 ms for each successive cycle until failure of stimulus capture. Incremental pacing protocols then began after achieving stable microelectrode impalement. These consisted of cycles of regular pacing, each of 100 stimulations. They began with a BCL of 130 ms that was then decremented by 5 ms for each subsequent cycle. These were repeated until the heart entered into 2:1 block or sustained arrhythmia.

### Data and statistical analysis

Data captured by the Spike2 software package (Cambridge Electronic Design) was analysed using a custom‐written program using the python programming language. Alternans was defined as an occurrence of alternating beat‐to‐beat changes in the value of a parameter such that the direction of the change oscillates for at least 12 successive action potentials. Statistical analysis was carried out using the R programming language (R Core Team, 2015) and plots with the grammar of graphics package (Wickham, 2009). All data are expressed as means ± SD, and a *P* value of < 0.05 was taken to be significant. Different experimental groups were compared with a two‐factor ANOVA. *F* values that were significant for interactive effects prompted *post hoc* testing with Tukey's honest significant difference testing. If single comparisons were made, Student's two‐tailed *t* test was used to compare significance. Categorical variables were compared using Fisher's exact test. Kaplan–Meier estimates were compared with the log rank test.

## Results

### Aged *Pgc1β* ^−/−^ hearts develop a pro‐arrhythmic phenotype

**Figure 1:**
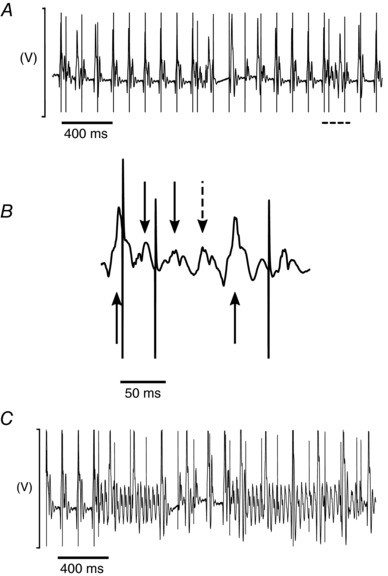
Electrocardiographic features of pro‐arrhythmic phenotypes in *Pgc‐1β* ^−/−^ atria *A*, typical ECG recordings from S1S2 protocols, demonstrating premature atrial complexes; dashed section is shown in *B*, with an expanded time base. Continuous downward‐pointing arrows denote atrial complexes as a result of stimulation. The dashed arrow indicates a premature atrial complex with no preceding stimulation spike. Continuous upward‐pointing arrows denote ventricular complexes. *C* illustrates atrial tachycardia in response to an S2 stimulus.

Electrocardiographic recordings were first made through the S1S2 protocol. Extrasystolic (S2) stimuli were interposed at successively shorter intervals following trains of eight regular (S1) stimuli applied at a 125 ms basic cycle length. This explored for the presence and the frequency of arrhythmic phenotypes in the intact *ex vivo* Langendorff‐perfused hearts. Figure 1 shows typical ECG recordings from aged *Pgc1β* ^−/−^ hearts at a slow time base during an S1S2 stimulation protocol. These include episodes of premature atrial complexes following successive S2 stimuli and a short run of atrial tachycardia captured during a typical stimulus train at the end of an S1S2 protocol (Fig. 1 *A*). On an expanded time base, these could be characterized by spontaneous atrial P waves (dashed arrow) in contrast to the paced P waves (continuous downward‐pointing arrows) following imposed pacing spikes (Fig. 1 *B*, arrowed) between successive ventricular complexes (upward‐pointing arrows). Some protocols also elicited runs of atrial tachycardia (Fig. 1 *C*). The S1S2 interval at the onset of failure of stimulus capture made it possible to determine the AERP corresponding to the specific (8 Hz) pacing rate. A comparison of AERPs obtained from the S1S2 protocol in young WT (24.8 ± 5.8 ms), aged WT (28.8 ± 6.1 ms), young *Pgc1β* ^−/−^ (29.3 ± 3.8 ms) and aged *Pgc1β* ^−/−^ hearts (30.1 ± 8.9 ms) demonstrated no significant differences between groups and provided indications of the extent to which BCLs could be decreased in the succeeding incremental pacing experiments.

**Figure 2:**
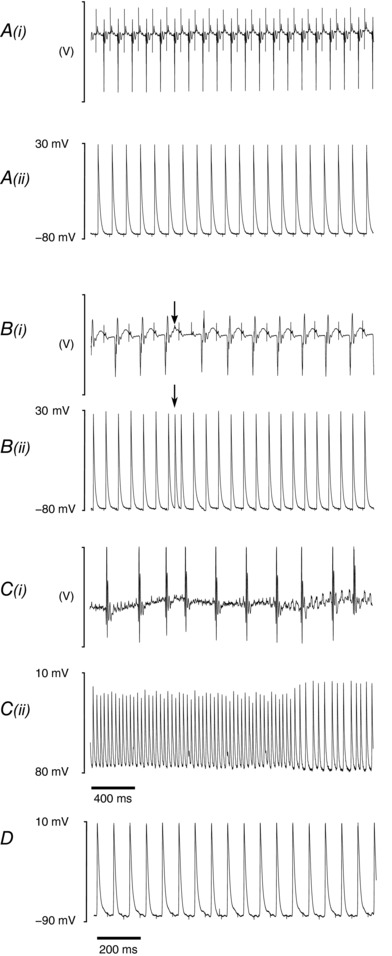
Electrocardiographic and intracellular recordings during incremental pacing *A*, typical recordings from incremental pacing protocols illustrating steady‐state ECG recordings (*i*) from a wild‐type (WT) heart as well as the corresponding intracellular action potential (AP) recordings (*ii*). Ectopic activity (arrow) from an aged *Pgc1β* ^−/−^ heart is shown in *B*, with the corresponding ECG (*i*) and intracellular AP recordings (*ii*). *C*, evidence of atrial fibrillation in the ECG (*i*) recording of an aged *Pgc1β* ^−/−^ heart, with concurrent intracellular AP recording (*ii*). *D* illustrates AP duration alternans in an AP recording from a young WT heart.

We then performed simultaneous whole‐heart ECG recordings and intracellular AP measurements from single cardiomyocytes after achieving stable microelectrode impalements, with consistent stimulating and recording electrode positions. The intracellular AP recordings provided accurate measurements of AP characteristics related to AP initiation, activation and recovery. These included maximal AP upstroke rates (d*V*/d*t*_max_), AP latencies, defined as the time interval between the pacing spike of each AP and the maximal deflection, or peak, of the AP, AP durations at 90% recovery (APD_90_), and resting membrane potentials (RMPs). The measurements of d*V*/d*t*_max_ and RMPs would not have been available with the monophasic action potential electrode methods used on previous occasions (Sabir *et al*. 2007a, 2008a). The incremental pacing protocols applied cycles of 100 regular pacing stimuli at successively decremented BCLs. Figure 2 illustrates results of the subsequent incremental pacing procedure comparing ECG (*i*) and intracellular traces (*ii*) in conditions of regular activity (Fig. 2 *A*) and occurrences of premature atrial complexes (Fig. 2 *B*), atrial fibrillation (Fig. 2 *C*) and APD_90_ alternans (Fig. 2 *D*) in an aged *Pgc1β^−/−^* heart. Individual hearts subjected to incremental pacing could therefore display phenotypes that differed from the results of extrasystolic (S2) stimuli.

**Figure 3:**
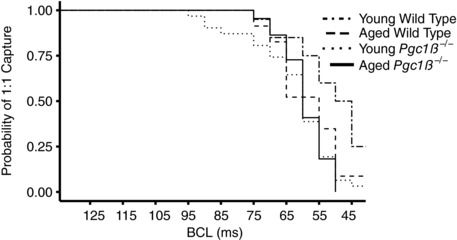
Kaplan–Meier plot of probability of 1:1 capture as basic cycle length (BCL) is varied for each experimental group

The cycles of incremental pacing continued with decreasing BCLs until the onset of either 2:1 capture or arrhythmia was reached. Kaplan–Meier curves plotting the probability of 1:1 capture of the groups as a function of BCL (Fig. 3) demonstrated a progressive reduction in the number of hearts continuing to show 1:1 capture at BCLs shorter than ∼70 ms. This would reflect their refractory properties at the steady‐state pacing frequencies close to this cut‐off. The statistical analysis to follow will therefore analyse data for parameters at BCLs no shorter than ∼50 ms. A log rank test confirmed that the survival curves were in fact significantly different (*P* = 0.0028). Young WT hearts showed fall‐offs at shorter BCLs than in the remaining groups and thus could be paced at higher frequencies than the other hearts, including aged WT hearts.

Table 1 summarizes the incidences of arrhythmic phenomena, whether in the form of atrial tachycardia or ectopic atrial deflections, through both pulse protocols. These together suggested a more marked pro‐arrhythmic phenotype in aged *Pgc1β* ^−/−^ hearts than in the remaining groups. With the S1S2 pulse protocol, the incidence of ectopic deflections was similar between groups, but the incidence of atrial tachycardias was greater in aged *Pgc1β* ^−/−^ hearts than in young WT, aged WT or young *Pgc1β* ^−/−^ hearts, which all showed similar incidences of atrial tachycardias (*P* = 0.043). Hearts displaying arrhythmias with extrasystolic (S2) provocation often failed to show arrhythmias on incremental pacing, and a relatively small number (three) of hearts showed arrhythmia amongst the experimental groups. The incremental pacing protocol thus resulted in few incidences of atrial tachycardia. Nevertheless, incidences of ectopic deflections were then greater in aged *Pgc1β* ^−/−^ hearts than in young WT, aged WT and young *Pgc1β* ^−/−^ hearts (*P* = 0.041).

**Table 1:** Incidences of atrial arrhythmic events during programmed electrical stimulation and incremental pacing in young and aged wild‐type (WT) and *Pgc1β* ^−/−^ hearts

	S1S2 protocol		Incremental pacing	
Experimental group	Atrial tachycardia	Ectopic events	Atrial tachycardia	Ectopic events
Young WT	3/20	8/20	0/20	2/20
Aged WT	4/23	8/23	1/23	4/23
Young *Pgc1β* ^−/−^	5/23	13/23	1/23	4/23
Aged *Pgc1β* ^−/−^	11/22*	8/22	1/22	10/22*

Data are represented as arrhythmic over total number studied. ^*^ *P* < 0.05 on Fisher exact testing.

### Altered atrial AP characteristics in young and aged *Pgc1β* ^−/−^ hearts

**Figure 4:**
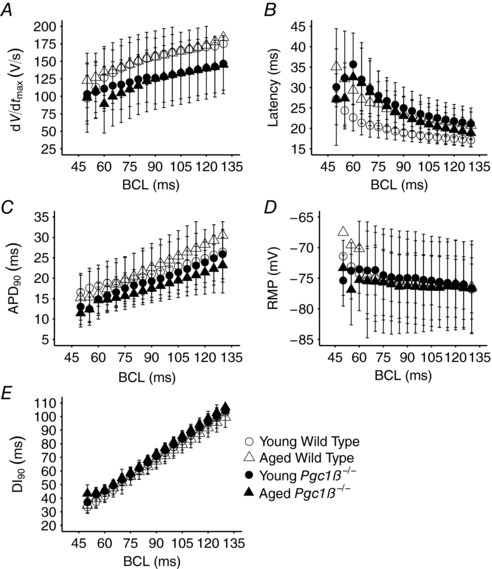
Dependence of maximal rate of action potential depolarization (d*V*/d*t*_max_; *A*), AP latency (*B*), AP durations to 90% recovery (APD_90_; *C*), resting membrane potential (RMP; *D*) and diastolic interval (DI_90_; *E*) on basic cycle length (BCL) in young and aged WT and *Pgc1β* ^−/−^ hearts Number of replicates: young WT, *n* = 20; young *Pgc1β* ^−/−^, *n* = 23; aged WT, *n* = 22; and aged *Pgc1β* ^−/−^, *n* = 22.

Figure 4 summarizes the activation and recovery characteristics of APs obtained through the incremental pacing procedures. It illustrates the corresponding alterations in d*V*/d*t*_max_ (Fig. 4 *A*), AP latencies (Fig. 4 *B*), APD_90_ (Fig. 4 *C*), RMP (Fig. 4 *D*) and diastolic intervals intervening between the attainment of 90% of AP recovery and the peak of the subsequent action potential (DI_90_; Fig. 4 *E*) with alterations in BCL in young and aged *Pgc1β* ^−/−^ and WT hearts. These parameters varied in a approximately linear manner with BCL, and their overall magnitudes could be compared by the areas beneath their curves. These are summarized in Table 2 which, in addition to showing results from the individual experimental groups of young and aged WT and *Pgc1β* ^−/−^ hearts, also provides the results of grouping all aged, young as well as WT and *Pgc1β* ^−/−^ atria for the statistical analysis. Two‐way ANOVA demonstrated that the *Pgc1β* ^−/−^ hearts displayed decreased d*V*/d*t*_max_ (*P* = 0.000020) and lower APD_90_ values (*P* = 0.00018) compared with WT hearts; there were no variations in RMP between groups. Genotype and age exerted significant interacting effects on DI_90_ and AP latency values (*P* = 0.0081, *P* = 0.043, respectively). *Post hoc* tests did not reveal further statistically significant differences.

**Table 2:** Areas under the curves of action potential (AP) parameters with respect to basic cycle length

Parameter	WT	Young WT	Aged WT	*Pgc1β* ^−/−^	Young *Pgc1β* ^−/−^	Aged *Pgc1β* ^−/−^	Young	Aged
d*V*/d*t*_max_ (mV)	11,765.1 ± 2574.6****	12,049.22 ± 2824.5	11,518.04 ± 2831.4	8979.36 ± 3014.5****	8945.29 ± 2891.4	9027.37 ± 3225.0	10,162.52 ± 3198.7	10,300.38 ± 3050.0
APD_90_ (ms^2^)	1702.79 ± 497.9***	1703.66 ± 537.3	1702.03 ± 474.4	1364.53 ± 309.4***	1396.49 ± 314.0	1319.49 ± 292.9	1516.95 ± 423.2	1515.01 ± 437.7
DI_90_ (ms^2^)	5292.48 ± 607.2††	5486.3 ± 596.2	5123.95 ± 578.4	5300.77 ± 610.9††	5160.93 ± 546.1	5497.81 ± 474.8	5288.52 ± 645.4††	5306.73 ± 557.8††
AP Latency (ms^2^)	1721.39 ± 374.3†	1638.49 ± 318.2	1793.47 ± 408.8	1831.90 ± 342.3†	1890.34 ± 376.0	1753.00 ± 252.0	1779.43 ± 366.2†	1774.65 ± 347.5†
RMP (mV ms)	−5374.71 ± 623.4	−5492.91 ± 715.0	−5271.93 ± 530.6	5226.35 ± 748.0	−5075.36 ± 800.2	−5439.1 ± 563.9	−5239.11 ± 797.5	−5353.66 ± 547.0

Each symbol represents statistical results from ANOVA. ^***^ *P* < 0.001 and ^****^ *P* < 0.0001 for independent effects of genotype. ^†^ *P* < 0.05 and ^††^ *P *< 0.01 for interacting effects of genotype and age. Number of replicates: young WT, *n* = 20; young *Pgc1β* ^−/−^, *n* = 23; aged WT, *n* = 22; and aged *Pgc1β* ^−/−^, *n* = 22. AP, action potential; d*V*/d*t*_max_, maximum AP upstroke rate; APD_90_, time to 90% AP recovery; DI_90_, diastolic interval from 90% AP recovery; RMP, resting membrane potential.

### Reduced temporal heterogeneities in atrial AP characteristics in aged *Pgc1β* ^−/−^ hearts

**Figure 5:**
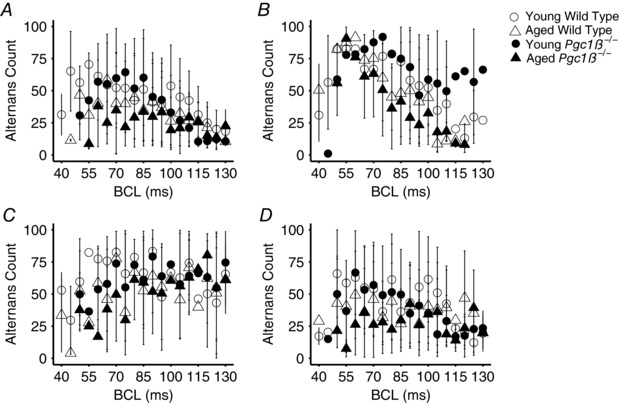
Incidence of alternans out of 100 beats at each BCL in the activation variables of maximal rate of AP depolarization, d*V*/d*t*_max_ (*A*) and AP latency (*B*), and the recovery variables of APD_90_ (*C*) and RMP (*D*) in young WT (open circles), young *Pgc1β* ^−/−^ (filled circles), old WT (open triangles) and *Pgc1β* ^−/−^ hearts (filled triangles) Number of replicates: young WT, *n* = 20; young *Pgc1β* ^−/−^, *n* = 23; aged WT, *n* = 22; and aged *Pgc1β* ^−/−^, *n* = 22.

Instabilities in characteristics of successive APs, often taking the form of episodes of alternans, presage major ventricular arrhythmias in clinical situations. They have been described in experimental conditions as alternating variations in temporal properties of AP excitation and/or recovery that occur with varying heart rates in analyses of pro‐arrhythmic tendencies associated with ventricular arrhythmogenesis (Sabir *et al*. 2007a, 2008a). We sought to analyse whether such phenomena are important in atrial arrhythmogenesis. Figure 5 summarizes the incidences of such alternans in the activation parameters d*V*/d*t*_max_ (Fig. 5 *A*) and AP latency (Fig. 5 *B*) and the recovery parameters APD_90_ (Fig. 5 *C*) and RMP (Fig. 5 *D*) in young and aged WT and *Pgc1β* ^−/−^ hearts through the incremental pacing protocol. Overall incidences of alternans were assessed by summing the individual incidence of alternans at each BCL. The different groups showed similar distributions in the occurrence of alternans at different BCLs. However, statistical comparisons of the overall incidences of alternans throughout the entire range of BCLs indicated that *Pgc1β* ^−/−^ hearts have reduced incidences and durations of alternans episodes.

Thus, two‐way ANOVA demonstrated that there were no significant effects of genotype on the incidence of alternans in d*V*/d*t*_max_, APD_90_, AP latency or RMP. Ageing independently reduced the incidence of alternans in aged compared with young hearts (d*V*/d*t*_max_ 24 ± 19.9 *versus* 39 ± 19.7 beats, *P* = 0.0013; APD_90_, 37 ± 19.9 *versus* 50 ± 19.7 beats, *P* = 0.0051; AP latency, 53 ± 19.9 *versus* 68 ± 19.7 beats, *P* = 0.00045; and RMP, 30 ± 13.2 *versus* 40 ± 13.1 beats; *P* = 0.0011). Age and genotype exerted interacting effects on the incidence of AP latency alternans (*P* = 0.032). *Post hoc* testing demonstrated less AP latency alternans in aged than young *Pgc1β* ^−/−^ hearts (47 ± 23.5 *versus* 71 ± 14.4 beats, *P* = 0.00041) and in aged *Pgc1β* ^−/−^ than young WT hearts (47 ± 23.5 *versus* 64 ± 22.4 beats, *P* = 0.036).

**Figure 6:**
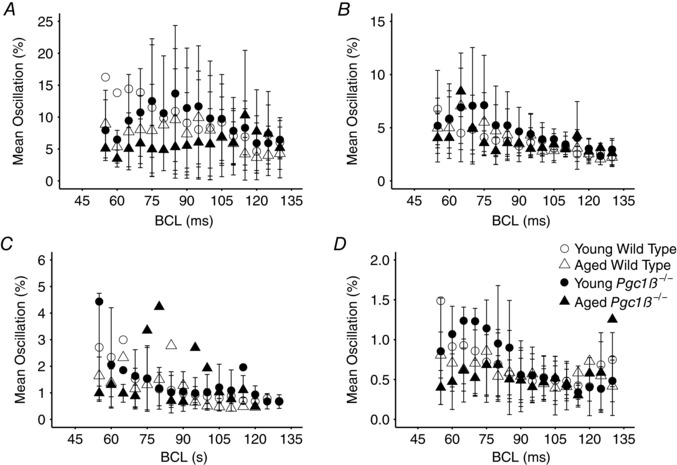
Magnitude of alternans as a percentage of the previous beat at each BCL for d*V*/d*t*_max_ (*A*), AP latency (*B*), APD_90_ (*C*) and RMP (*D*) Number of replicates: young WT, *n* = 20; young *Pgc1β* ^−/−^, *n* = 23; aged WT, *n* = 22; and aged *Pgc1β* ^−/−^, *n* = 22.

Likewise, two‐way ANOVA demonstrated that the magnitude of d*V*/d*t*_max_, APD_90_ and RMP (although not AP latency) alternans, reflected in the areas under the respective curves, was smaller in aged than young hearts (*P* = 0.037, *P* = 0.038, *P* = 0.052 and *P* = 0.066, respectively; Fig. 6). There were no effects of genotype or interacting effects of genotype and age together on the overall magnitudes of oscillation. The total numbers of episodes of APD_90_, AP latency or RMP alternans were indistinguishable between groups, whilst *Pgc1β* ^−/−^ hearts showed fewer episodes of d*V*/d*t*_max_ alternans than WT hearts (8.54 ± 5.0 *versus* 13.37 ± 9.5 episodes of alternans, *P* = 0.0021) and aged hearts fewer episodes of d*V*/d*t*_max_ alternans than young hearts (9.24 ± 7.5 *versus* 12 ± 7.5 episodes of alternans, *P* = 0.030).

Maximal durations of individual episodes of d*V*/d*t*_max_, AP latency, APD_90_ or RMP alternans were all shorter in aged than young hearts (d*V*/d*t*_max_, 44 ± 66.3 *versus* 139 ± 177.0 beats, *P* = 0.0015; AP latency, 140 ± 126.0 *versus* 352 ± 183.6, *P* = 2.7 × 10^−8^; APD_90_, 247 ± 325.0 *versus* 390 ± 393.4 beats, *P* = 0.049; and RMP, 64 ± 66.3 *versus* 136 ± 131.1 beats, *P* = 0.0011). Although they were affected by interacting effects of age and genotype (*P* = 0.036), *post hoc* testing demonstrated that these maximal durations were shorter in both aged *Pgc1β* ^−/−^ and aged WT hearts than young *Pgc1β* ^−/−^ hearts (98 ± 79.7 *versus* 379 ± 167.8 beats, *P* < 0.00001, and 180 ± 150.0 *versus* 379 ± 167.8 beats, *P* = 0.00029, respectively). Furthermore, aged *Pgc1β* ^−/−^ hearts also showed shorter maximal durations of AP latency alternans than young WT hearts (98 ± 79.7 *versus* 311 ± 210.2 beats, *P* = 0.00061).

Finally, alternans simultaneously involving different AP characteristics could involve alternating high/low AP latencies or reduced/increased d*V*/d*t*_max_ coinciding with or, in the more pro‐arrhythmic pattern, occurring out of phase with higher/lower APD_90_ values. However, reduced frequencies of simultaneous d*V*/d*t*_max_ and APD_90_ alternans occurred in aged compared with young hearts (14.91 ± 16.1 *versus* 27.74 ± 19.9%, *P* = 0.00043) and *Pgc1β* ^−/−^ compared with WT hearts (17.77 ± 2.57 *versus* 26.59 ± 3.23%, *P* = 0.024). Reduced and similar proportions of out‐of‐phase alternans occurred in *Pgc1β* ^−/−^ hearts compared with WT hearts (7.83 ± 9.3 *versus* 19.30 ± 30.0%; *P* = 0.010) and aged compared with young hearts, respectively (*P* = 0.070). Likewise, reduced frequencies of simultaneous APD_90_ and AP latency alternans occurred in aged compared with young hearts (25.67 ± 16.5 *versus* 45.53 ± 15.9%, *P* = 0.082 × 10^−5^), which showed fewer beats of the more pro‐arrhythmic alternans pattern (132 ± 152.6 *versus* 334 ± 262.3 beats; *P* = 0.00017).

### Spatiotemporal representations of AP excitation in *Pgc1β* ^−/−^ and WT hearts

**Figure 7:**
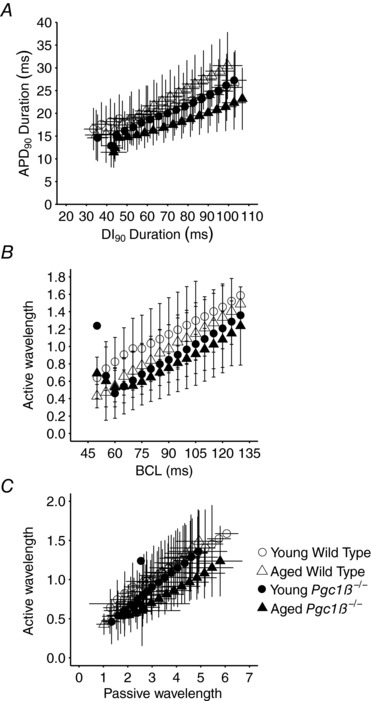
Restitution plots of APD_90_ against DI_90_ (*A*) and of active AP wavelength (*B*) and passive wavelength (*C*) observed at different BCLs through the incremental pacing procedure in young and old WT and *Pgc1β* ^−/−^ hearts Number of replicates: young WT, *n* = 20; young *Pgc1β* ^−/−^, *n* = 23; aged WT, *n* = 22; and aged *Pgc1β* ^−/−^, *n* = 22.

Previous reports in murine ventricles correlated variations in the dependence of AP recovery upon BCL with instabilities in the form of alternans. Restitution curves of APD_90_ against BCL, or diastolic intervals (DI_90_) from 90% action potential recovery (Sabir *et al*. 2008a,b), then associated pro‐arrhythmic instabilities with increasing limiting slopes in APD_90_ *versus* DI_90_ plots with shortening DI_90_ (Matthews *et al*. 2012). Attainment of unity gradient presaged waxing AP alternans and arrhythmia. In contrast, results in Fig. 7 *A* implicated neither genotype (two‐way ANOVA: *P* = 0.12) nor age (*P* = 0.15) in independently influencing these limiting slopes. These factors did interact (*P* = 0.0001), but this gave reduced slopes in aged *Pgc1β^−/−^* atria (Tukey's tests: aged *Pgc1β* ^−/−^ *versus* aged WT, *P* = 0.001; aged WT *versus* young WT, *P* = 0.001), consistent with the observed paradoxically decreased incidences and durations of alternans in aged and *Pgc1β* ^−/−^ hearts and contrasting with their increased arrhythmogenic properties.

In contrast, analysis of spatial representations of action potential activation, as opposed to the temporal characteristics of AP recovery considered above, replicated the arrhythmic phenotypes in terms of the accompanying electrophysiological abnormalities particularly in aged *Pgc1β* ^−/−^ hearts. Here, the AP travelling waves were represented in terms of wavelengths of excited tissue undergoing APs (λ) and resting wavelengths (λ_0_) of the succeeding tissues that had completed their subsequent AP recovery. These λ and λ_0_ terms were obtained by multiplying 1/(AP latency), reflecting conduction velocity, by the corresponding APD_90_ or DI_90_ values, respectively (Matthews *et al*. 2013b). These were then compared in young and aged WT and *Pgc1β* ^−/−^ atria through the different BCLs examined. Areas under plots of λ against BCL (Fig. 7 *B*) then demonstrated that the *Pgc1β* ^−/−^ as opposed to the WT genotype, but not age, independently (two‐way ANOVA: *P* = 0.6 × 10^−4^ and 0.14, respectively) reduced λ. Additional, interacting effects (*P* = 0.048) through the range of explored BCLs, were reflected in the shorter λ values in both young (Tukey's test: *P* = 0.0001) and aged *Pgc1β* ^−/−^ (Tukey's test: *P* = 0.0008) compared with young WT atria. Likewise, λ values from the experimental groups all declined and converged with declining λ_0_ and with shortening BCL, as previously reported (Matthews *et al*. 2013*a*; Ning *et al*. 2016b; Fig. 7 *C*). Nevertheless, λ values in aged *Pgc1β* ^−/−^ hearts consistently fell below those in remaining groups (*n* = 14 points, sign test, *P* < 0.01). Accordingly, areas beneath the curves reflected significantly greater λ at the longer (85–130 ms; two‐way ANOVA: *P* = 0.042) but not the shorter (<85 ms) BCLs, resulting from interacting effects of age and *Pgc1β* ^−/−^ genotype (Matthews *et al*. 2013*a*; Ning *et al*. 2016b).

## Discussion

Both age and energetic dysfunction are known risk factors for atrial fibrillation (Go *et al*. 2001; Menezes *et al*. 2013). The experimental basis for this effect was studied in young and aged Pgc1‐deficient hearts used in previous biochemical studies of mitochondrial dysfunction. Pgc1 upregulates mitochondrial function and cellular energy homeostasis (Lin *et al*. 2005; Finck & Kelly, 2006), acting on genes involved in fatty acid oxidation and electron transport (Arany *et al*. 2005). Both ageing WT (Froehlich *et al*. 1978; Lakatta & Sollott, 2002; Hatch *et al*. 2011; Yang *et al*. 2015) and energetically deficient *Pgc1β* ^−/−^ hearts show abnormalities in a range of cellular mechanisms with potential electrophysiological consequences for arrhythmia. Their increased production of reactive oxygen species (Grivennikova *et al*. 2010) modifies maximal voltage‐dependent Na^+^ and K^+^ current (Wang *et al*. 2004; Liu *et al*. 2010), sarcolemmal K_ATP_ channel function, Na^+^ and Ca^2+^ channel inactivation, and late Na^+^ current and ryanodine receptor function (Huang, 2017). The associated ATP/ADP depletion opens sarcolemmal ATP‐sensitive K^+^ channels (sarcK_ATP_; Akar & O'Rourke, 2011). Additional abnormalities in Ca^2+^ homeostasis and delayed after‐depolarization events potentially initiate pro‐arrhythmic triggering activity (Gurung *et al*. 2011). These mechanisms could in turn potentially alter cell–cell coupling (Smyth *et al*. 2010), AP conduction (Liu *et al*. 2010), repolarization and refractoriness (Wang *et al*. 2004), as well as predispose to alternans and Ca^2+^‐mediated pro‐arrhythmic triggering phenomena (Terentyev *et al*. 2008).

Recent studies have reported both compromised heart rate responses to adrenergic stimulation (Lelliott *et al*. 2006) and pro‐arrhythmic ventricular phenotypes in intact perfused murine *Pgc1β* ^−/−^ hearts (Gurung *et al*. 2011). However, the corresponding atrial electrophysiological AP or arrhythmic phenotypes have not been investigated. In the present study, we applied cellular electrophysiological recordings in cardiomyocytes within intact, normally functioning Langendorff‐perfused hearts as opposed to isolated cardiomyocytes. We thereby determined steady‐state arrhythmic and related restitution properties of murine atrial tissue for the first time.

Our experimental approach permitted simultaneous study of atrial arrhythmic properties of the whole heart and electrophysiological properties of single atrial cells. Aged *Pgc1β* ^−/−^ hearts demonstrated a significantly greater incidence of arrhythmogenic phenotypes compared with all the remaining groups. The stimulation procedures that applied extrasystolic S2 stimuli resulted in a higher incidence of atrial tachycardias, whereas those applying incremental increases in steady heart rates resulted in a higher incidence of ectopic atrial events. The latter suggests that atrial cardiomyocytes have a greater capacity for rapid pacing without producing pro‐arrhythmic phenomena.

The association of the arrhythmic phenotype with alterations in the corresponding AP characteristics was then examined. Of the measured AP parameters, resting membrane potentials remained uniform throughout all experimental groups, consistent with clinical findings in atrial fibrillation (Bosch *et al*. 1999). The statistically most noticeable alterations involved compromised AP activation, which has been implicated in arrhythmic substrate on previous occasions (Huang *et al*. 2012; King *et al*. 2013a; Huang, 2017). Thus, *Pgc1β* ^−/−^ atria displayed decreased d*V*/d*t*_max_ compared with WT atria, in the absence of statistical effects of age or interactions between age and genotype. The d*V*/d*t*_max_ correlates with peak Na^+^ current, which in turn markedly influences AP conduction velocity to an extent dependent upon the conductivity between cells (Hunter *et al*. 1975; Hondeghem & Katzung, 1977; Usher‐Smith *et al*. 2006; Fraser *et al*. 2011). Although young and aged *Pgc1β* ^−/−^ atria did not demonstrate significant differences in d*V*/d*t*_max_, AP latency measurements reflecting conduction velocity were influenced by interactions between age and genotype. They therefore account for the more marked pro‐arrhythmic phenotype in the aged than the young *Pgc1β* ^−/−^ atria.

These findings are compatible with hypotheses relating reduced d*V*/d*t*_max_ and increased AP latency to decreased peak Na^+^ current in the conditions of age‐dependent mitochondrial dysfunction in *Pgc1β* ^−/−^ hearts (Hondeghem & Katzung, 1977; Usher‐Smith *et al*. 2006; Fraser *et al*. 2011). Established reports associate metabolic insufficiency with reduced Na^+^ channel function in a number of circumstances. First, in addition to reduced provision of ATP, disrupted mitochondrial activity increases production of reactive oxygen species (Manning *et al*. 1984; Fosset *et al*. 1988; Faivre & Findlay, 1990) and perturbs cytosolic NAD^+^/NADH. Both factors are implicated in altered Na^+^ channel function in metabolically stressed cardiomyocytes (Liu *et al*. 2009) and are rescued by the mitochondrial reactive oxygen species scavenger mitoTEMPO (Liu *et al*. 2010).

Second, *Pgc1β* ^−/−^ cardiomyocytes show altered Ca^2+^ homeostasis manifest in abnormal diastolic Ca^2+^ transients (Gurung *et al*. 2011). Other murine models show an altered Ca^2+^ homeostasis implicated in slowed AP conduction accompanying reduced d*V*/d*t*_max_ (Zhang *et al*. 2013). These were attributed to compromised Na^+^ currents resulting from (i) reduced Na_V_1.5 expression (Ning *et al*. 2016) and/or (ii) acute effects upon Na_V_1.5 function (King *et al*. 2013b,c). These observations were reported in *RyR2‐P2328S*/*P2328S* cardiomyocytes, which likewise show abnormal Ca^2+^‐handling phenotypes (Gurung *et al*. 2011). They also follow acute manipulations in Ca^2+^ homeostasis by caffeine or Epac‐mediated RyR2 agonism or cyclopiazonic acid‐mediated Ca^2+^‐ATPase antagonism (King *et al*. 2013b; Li *et al*. 2017). Patch‐clamped WT cardiomyocytes likewise show acutely reduced or increased Na^+^ current and d*V*/d*t*_max_, with respective increases in, or sequestration of, the pipette [Ca^2+^] (Casini *et al*. 2009).

Comparison of the present findings of reduced d*V*/d*t*_max_ and increased AP latency in cardiomyocytes in intact tissue with the normal or even enhanced Na^+^ currents in *Pgc1β* ^−/−^ cardiomyocytes subject to patch‐clamping involving Ca^2+^ chelation by intrapipette BAPTA (Gurung *et al*. 2011) are compatible with a mechanism of action involving the latter, acute effects, of altered Ca^2+^ homeostasis upon membrane excitability. This could involve Ca^2+^–Na_v_1.5 interactions involving direct Ca^2+^–Na_V_1.5 binding at an EF hand motif close to the Na_V_1.5 C‐terminal (Wingo *et al*. 2004) or indirect Ca^2+^ binding involving additional ‘IQ’ domain binding sites for Ca^2+^–calmodulin in the Na_V_1.5 C‐terminal region (Mori *et al*. 2000; Wagner *et al*. 2011; Grandi & Herren, 2014). If so, these findings in a model for metabolic disturbance provide a further example of potentially important effects of intracellular Ca^2+^ homeostasis on arrhythmic substrate through altering AP propagation as a result of acute effects upon Na^+^ channel function.

The electrophysiological alterations of atrial cardiomyocytes presented in this study led to investigations to distinguish mechanisms for the resulting arrhythmic substrate at the tissue level (Martin *et al*. 2012), relating these to previous reports from monogenic murine arrhythmic models. First, arrhythmic syndromes primarily attributed to repolarization abnormalities have been exemplified by murine *Scn5a*+/*∆KPQ* hearts. Arrhythmic substrate was there associated with altered relationships between APD_90_ and effective refractory period with varying diastolic interval (DI_90_; Nolasco & Dahlen, 1968; Sabir *et al*. 2008a; Matthews *et al*. 2010, 2012) increasing the steepness of restitution curve plots of APD_90_ against DI_90_ at short BCLs. The consequent APD_90_ instabilities would increase frequencies and amplitudes of alternans, culminating in arrhythmic substrate (Huang, 2017).

However, the present findings did not implicate such alternans in the arrhythmic instability in aged *Pgc1β* ^−/−^ atria. Atrial alternans was observed in AP trains during incremental steady‐state pacing. However, its incidence was not increased by age or *Pgc1β* ^−/−^ genotype. Indeed, aged atria showed decreased incidences of alternans compared with young hearts. The *Pgc1β* ^−/−^ and aged atria showed fewer episodes of alternans than WT and young atria, respectively. Furthermore, it was the restitution curves of aged *Pgc1β* ^−/−^ hearts that showed the most reduced limiting slopes. These findings parallel previous reports in the vagally induced model of canine AF that likewise showed paradoxically less alternans and a flatter restitution slope than in the non‐arrhythmic control state (Lu *et al*. 2011). Atrial *Pgc1β* ^−/−^ cardiomyocytes thus showed greater capacity to follow rapid pacing without producing pro‐arrhythmic alternans phenomena. This would be compatible with their shorter APDs reflecting more rapid AP recoveries, despite their more prolonged activation processes.

Second, other arrhythmic syndromes have been contrastingly attributed to altered AP conduction, exemplified by murine *Scn5a* ^+/−^ hearts (Martin *et al*. 2012). This modifies the spatial extent and homogeneity of the travelling waves of AP excitation, or the quiescence that follows it. These were quantified by active wavelengths (λ) derived from conduction velocity [reflected by 1/(AP latency)] and APD terms (given by APD_90_; Matthews *et al*. 2013), and resting wavelengths (λ_0_) comprising DI_90_ and AP latency terms (Matthews *et al*. 2013; Ning *et al*. 2016). Larger λ values reduce likelihoods that areas of depolarization and repolarization coincide at tissue heterogeneities, increasing the safety factor that ensures continued undisrupted propagation of the travelling wave (Weiss *et al*. 2005). Conversely, decreased λ values increase such likelihoods and those of the consequent wave break‐ups into multiple wavelets, scroll‐waves (Davidenko *et al*. 1995; Zaitsev *et al*. 2000; Pandit & Jalife, 2013) and further wavebreaks disrupting the AP conduction pathways (Spector, 2013). Clinical observations correspondingly associate short AP wavelengths with AF inducibility and maintenance (Hwang *et al*. 2015), particularly in AF patients (Padeletti *et al*. 1995).

The present analysis indeed demonstrated that in plots of λ against either BCL or λ_0_, *Pgc1β* ^−/−^ and, particularly, aged *Pgc1β* ^−/−^ atria consistently gave shorter λ in direct parallel with their pro‐arrhythmic phenotype. Altered AP conduction with its consequences for its spatiotemporal properties thus constitutes a potential mechanism for the atrial arrhythmic changes associated with age and energetic compromise reported here.

## Additional information

### Competing interests

None declared.

### Author contributions

C.L.‐H.H., H.V. and S.A. were involved in conception and design of the work, acquisition, analysis and interpretation of data, and drafting and critical revision of the manuscript. J.A.F. was involved in conception and design and drafting the work. K.J. was involved in acquisition, analysis and interpretation of data and critical revision. All authors approved the final version of the manuscript and agree to be accountable for all aspects of the work in ensuring that questions related to the accuracy or integrity of any part of the work are appropriately investigated and resolved. All persons designated as authors qualify for authorship, and all those who qualify for authorship are listed.

### Funding

We thank the Wellcome Trust (105727/Z/14/Z), Medical Research Council (MR/M001288/1), British Heart Foundation (PG/14/79/31102 and PG/15/12/31280), Isaac Newton Trust–Wellcome Trust ISSF–University of Cambridge Joint Research Grants Scheme, Sudden Arrhythmic Death Syndrome (SADS) UK society, and the Fundamental Research Grant Scheme (FRGS/2014/SKK01/PERDANA/02/1; Ministry of Education, Malaysia) for their generous support.
